# Comprehensive Analysis of Antibodies Induced by Vaccination with 4 Kinds of Avian Influenza H5N1 Pre-Pandemic Vaccines

**DOI:** 10.3390/ijms21197422

**Published:** 2020-10-08

**Authors:** Nobuko Ohshima, Yoshitaka Iba, Ritsuko Kubota-Koketsu, Ayami Yamasaki, Keiko Majima, Gene Kurosawa, Daisuke Hirano, Shunji Yoshida, Mototaka Sugiura, Yoshizo Asano, Yoshinobu Okuno, Yoshikazu Kurosawa

**Affiliations:** 1 International Center for Cell and Gene Therapy, Research Promotion and Support Headquarters, Fujita Health University, Toyoake, Aichi 470-1192, Japan; ibayoshi@fujita-hu.ac.jp (Y.I.); gene@fujita-hu.ac.jp (G.K.); 2 Department of Innovation for Advanced Medicine, Center for Research Promotion and Support, Fujita Health University, Toyoake, Aichi 470-1192, Japan; aya.ipomoea@gmail.com (A.Y.); kei-majima@outlook.com (K.M.); 3 Clinical Research Section, Research Department, The Research Foundation for Microbial Disease of Osaka University, Suita, Osaka 565-0871, Japan; koketsu@biken.osaka-u.ac.jp; 4 Division of Innovation for Advanced Medicine, Institute for Comprehensive Medical Science, Fujita Health University, Toyoake, Aichi 470-1192, Japan; 5 Department of Academic Research Support Promotion Facility, Center for Research Promotion and Support, Fujita Health University, Toyoake, Aichi 470-1192, Japan; 6 Division of Rheumatology, Department of Internal Medicine, School of Medicine, Fujita Health University, Toyoake, Aichi 470-1192, Japan; d-hirano@fujita-hu.ac.jp (D.H.); yoshida@fujita-hu.ac.jp (S.Y.); 7 Department of Internal Medicine, School of Medicine, Fujita Health University, Toyoake, Aichi 470-1192, Japan; msugiura@fujita-hu.ac.jp; 8 Department of Pediatrics, School of Medicine, Fujita Health University, Toyoake, Aichi 470-1192, Japan; 9 Osaka Institute of Public Health, Osaka 537-0025, Japan; okuno@iph.osaka.jp

**Keywords:** antibodies, pre-pandemic vaccines, H5N1

## Abstract

Four kinds of avian-derived H5N1 influenza virus, A/Vietnam/1194/2004 (Clade 1), A/Indonesia/5/2005 (Clade 2.1), A/Qinghai/1A/2005 (Clade 2.2), and A/Anhui/1/2005 (Clade 2.3), have been stocked in Japan for use as pre-pandemic vaccines. When a pandemic occurs, these viruses would be used as vaccines in the hope of inducing immunity against the pandemic virus. We analyzed the specificity of antibodies (Abs) produced by B lymphocytes present in the blood after immunization with these vaccines. Eighteen volunteers took part in this project. After libraries of Ab-encoding sequences were constructed using blood from subjects vaccinated with these viruses, a large number of clones that encoded Abs that bound to the virus particles used as vaccines were isolated. These clones were classified into two groups according to the hemagglutination inhibition (HI) activity of the encoded Abs. While two-thirds of the clones were HI positive, the encoded Abs exhibited only restricted strain specificity. On the other hand, half of the HI-negative clones encoded Abs that bound not only to the H5N1 virus but also to the H1N1 virus; with a few exceptions, these Abs appeared to be encoded by memory B cells present before vaccination. The HI-negative clones included those encoding broadly cross-reactive Abs, some of which were encoded by non-V_H_1-69 germline genes. However, although this work shows that various kinds of anti-H5N1 Abs are encoded by volunteers vaccinated with pre-pandemic vaccines, broad cross-reactivity was seen only in a minority of clones, raising concern regarding the utility of these H5N1 vaccine viruses for the prevention of H5N1 pandemics.

## 1. Introduction

Since the direct bird-to-human transmission of highly pathogenic avian influenza (HPAI) H5N1 virus occurred in Hong Kong in 1997, such viruses have spread to countries in Asia, the Middle East, Africa, and Europe [1]. The introduction of mutations in hemagglutinin (HA)-encoding sequences of highly malignant avian H5N1 influenza viruses has the potential to endow HPAI with the ability to spread by human-to-human transmission, which could result in a pandemic [2]. It has been estimated that such a pandemic could result in the deaths of up to 350 million people while affecting many more, causing disruption to healthcare systems, the world economy, and society at large [3]. This estimation was based on the following assumption. Since few (if any) people on earth have experienced infection with H5N1 viruses, the general population does not have any immunity against these viruses. Furthermore, since the first infection with HPAI virus in 1997, these pathogens have spread to many countries and caused human infections [4]. The human fatality rate of HPAI has been estimated to be around 60%. 

As one of the measures against such future pandemics, four kinds of vaccine using avian-derived H5N1 viruses (including A/Vietnam/1194/2004 (Vie04) (Clade 1), A/Indonesia/5/2005 (Ind05) (Clade 2.1), A/Qinghai/1A/2005 (Qin05) (Clade 2.2), and A/Anhui/1/2005 (Anh05) (Clade 2.3)) have been stocked in Japan for possible use as pre-pandemic vaccines; these vaccines have been stocked in doses sufficient for the immunization of ten million people [5,6]. When a pandemic occurs, it will take a long time to prepare large amounts of vaccine using the pandemic-causing virus itself. Therefore, these pre-pandemic vaccines will be used instead of a vaccine against the causative virus, with the expectation that vaccination with these vaccine viruses will induce at least partial immunity against the pandemic pathogen. The implementation of this counterplan was initiated in 2006.

Given this situation, the present study was performed, from 2015 to 2018, as a national project supported by the Japanese government. The most important subject to be addressed in the present study was to examine the effectiveness of the stocks of pre-pandemic vaccine as countermeasures against a future pandemic virus. As a first step, we sought to analyze the cross-reactivity of the antibodies (Abs) induced by vaccination with these pre-pandemic vaccines. As a second step, we sought to compare the four kinds of stocked H5N1 vaccine viruses for their efficiency in inducing the production of broadly neutralizing Abs. Since the initiation of this study in 2015, the Qin05 (Clade 2.2) strain has been replaced with A/Guangdong/17SF003/2016 as a member of the pre-pandemic vaccine panel. Nonetheless, we report here all of our results, with the expectation that the data obtained in this study will provide valuable new information, notably including the ability of these vaccine viruses to induce novel broadly cross-reactive Abs.

## 2. Results

### 2.1. Isolation, from Volunteers Vaccinated with H5N1 Vaccine, of Abs That Bound to H5N1 Virus Particles

Eighteen volunteers took part in this project as blood donors to examine the effects of vaccination with four kinds of H5N1 strains. Each volunteer was vaccinated twice at a one-week interval with various combinations of H5N1 strains. One month later, 100 mL of blood was collected from each volunteer and mononuclear cells were isolated. Eighteen Ab libraries were constructed from mRNA of the separated cells by utilizing phage-display technology. After heavy (H)-chain libraries and light (L)-chain libraries were constructed separately, the two were combined, resulting in expression of the Fab proteins from large Ab libraries, with each library representing a total of >1 × 10^9^ individual clones. 

The individual libraries were screened by panning, employing as bait the virus particles that had been used in vaccination. After three or four rounds of panning, several hundred clones were isolated from each library, and the binding activity of these Abs for the virus particles was determined. As summarized in Table 1, a total of 2461 clones were shown to have affinity for H5N1 vaccine viruses.

We determined the sequence of the V_H_-encoding region of each of the clones and classified the sequences based on the following principles. Clones that encoded V_H_ regions with identical amino acid sequences were treated as the same clones. Then, V_H_-encoding germline genes used in the respective clones were assigned. Among the different clones, if the amino acid sequences of the complementarity-determining region 3 (CDR3) domains were the same among clones that utilized the same germline V_H_-encoding gene, we judged that these clones were originally derived from the same B cell and that any observed differences in amino acid sequences were the result of subsequent mutations. 

Alternatively, where multiple clones derived from identical V_H_-encoding germline genes encoded highly similar CDR3 amino acid sequences, a clone corresponding to the most frequently isolated sequence was subjected to further analyses as a representative of this class. Clones that exhibited binding of recombinant HA were selected for further characterization. Thus, 1993 clones corresponding to 206 different clones and 95 representative clones were subjected to further analyses (Supplementary Blot 1).

### 2.2. HI Activities

The HI activities of 95 representative clones were measured by using the Fab-PP form of the Ab and the H5N1 virus that had been used for isolation of the respective clone. Among the 95 candidates, 62 clones showed HI activity that was distributed between 0.75 and 100 µg/mL, as indicated in Table 2; these isolates were defined as HI-positive (*n* = 62). Clones that were isolated in the present study but did not show HI activity even at 100 µg/mL were judged to be HI-negative (*n* = 33). Thus, sixty percent of the isolated clones showed HI activity. The HI-positive clones and HI-negative clones were analyzed separately in subsequent experiments.

### 2.3. Analyses of HI-Positive Clones

#### 2.3.1. H5N1 Vaccine Strain Binding by the HI-Positive Clones

**Figure 1:**
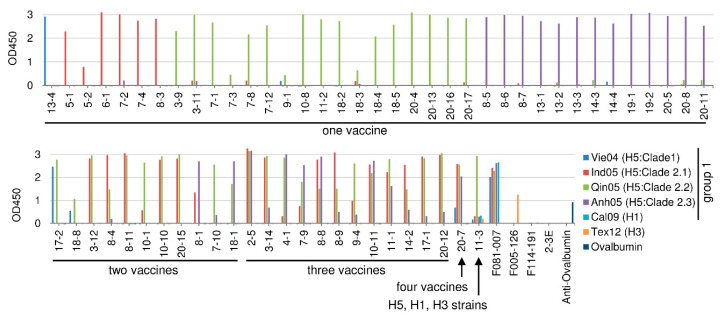
Binding activity of HI-positive clones. The binding activity of Fab-PP (produced from sixty-two clones) to vaccines of four H5N1, H1N1, and H3N2 strains was examined by ELISA. Clones F081-007 (anti-H1 and -H5 HA (hemagglutinin) antibody) and F005-126 (anti-H3 HA antibody) were used as positive controls. Clone 2-3E (anti-rotavirus antibody) was used as a negative control. The binding activity is shown as the optical density at 450 nm (OD450).

The 62 HI-positive clones were subjected to an analysis of strain specificity using the same assay as used for assessment of binding activity. Six kinds of vaccines (four H5N1 viruses (Vie04, Ind05, Qin05, Anh05), one H1N1 virus (Cal09), and one H3N2 virus (Tex12)) were used in this experiment. Figure 1 shows the results of these ELISAs. The strain specificity of the respective clones can be summarized as follows. The numbers of clones that bound to only one of the four H5N1 strains were as follows: Vie04 (Clade 1), 1 clone; Ind05 (Clade 2.1), 6 clones; Qin05 (Clade 2.2), 17 clones; Anh05 (Clade 2.3), 13 clones. The numbers of clones that bound to two of the four H5N1 viruses were as follows: Vie04 (Clade 1) and Qin05 (Clade 2.2), two clones; Ind05 (Clade 2.1) and Qin05 (Clade 2.2), six clones; Ind05 (Clade 2.1) and Anh05 (Clade 2.3), one clone; Qin05 (Clade 2.2) and Anh05 (Clade 2.3), two clones. The number of clones that bound to three of the four H5N1 viruses was as follows: Ind05 (Clade 2.1), Qin05 (Clade 2.2), and Anh05 (Clade 2.3), 12 clones. The number of clones that bound to all four of the four tested H5N1 viruses was as follows: two clones. Among the 62 HI-positive clones, Clone 11-3 showed binding to Cal09 (H1N1), albeit weakly. Clone 11-3 showed binding activity even to Tex12 (H3N2). Thus, HI-positive Abs showed binding activity to various combinations of H5N1 viruses.

#### 2.3.2. H5N1 Virus-Neutralizing Activity among the HI-Positive Clones

**Figure 2:**
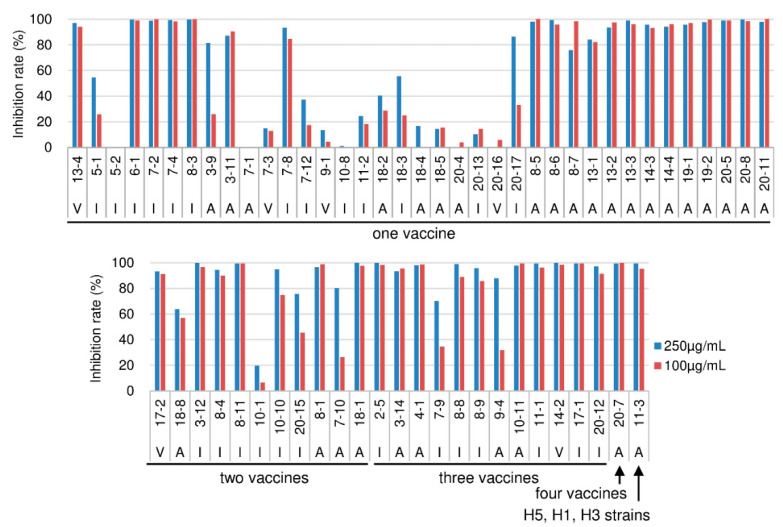
Neutralization activity of Fab-PP from HI-positive clones was measured by the focus reduction assay. Neutralization activity was tested at two antibody concentrations (250 µg/mL, blue bars; and 100 µg/mL, red bars). Three kinds of H5N1 viruses were tested, including V: Vie04 (Clade 1), I: Ind05 (Clade 2.1), and A: Anh05 (Clade 2.3); the strain used for each assay is indicated under the clone name. The neutralization activity is shown as the focus reduction rate (%) normalized to that observed in the absence of antibody.

Virus-neutralizing activity was measured directly by the focus reduction assay [7]. While the virus used in this experiment should have been the same type of virus as used in the vaccination, live Qin05 (Clade 2.2) virus was not available when we performed this study. Therefore, only three viruses (Vie04 (Clade 1), Ind05 (Clade 2.1), and Anh05 (Clade 2.3)) were tested. The degrees of focus reduction at the concentrations of 250 and 100 µg/mL of the Fab-PP form of Ab are indicated in Figure 2. Three clones (5-2, 7-1, and 10-8) did not show virus-neutralizing activity in this assay, but the binding activity of 5-2 was weak and the other two clones bound only to the Qin05 (Clade 2.2) virus (Figure 1).

Another nine clones (7-3, 9-1, 10-1, 11-2, 18-4, 18-5, 20-4, 20-13, and 20-16) showed low neutralizing activity. However, these clones also bound only to the Qin05 (Clade 2.2) virus; the sole exception was 10-1, which exhibited weak binding to Ind05 (Figure 1). Therefore, we concluded that virtually all (50/62; 80.6%) of the HI-positive clones showed virus-neutralizing activity.

#### 2.3.3. Cross-Reactivity against Recombinant HA Molecule among the HI-Positive Clones

**Figure 3:**
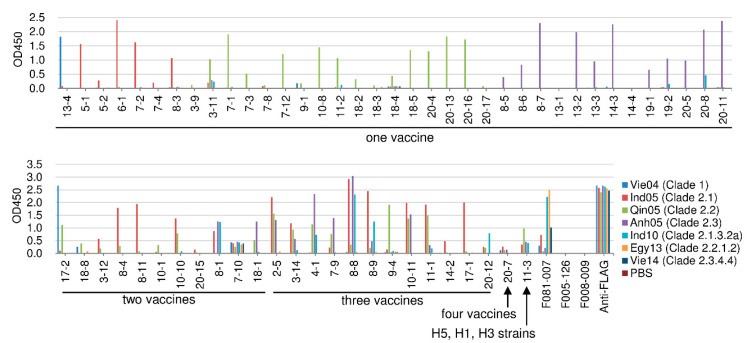
Binding activity of HI-positive clones to recombinant HA molecules derived from seven kinds of H5N1 viruses. Binding was measured by ELISA. F081-007 is an anti-H1 and -H5 HA antibody that was used as a positive control. F005-126 (an anti-H3 HA antibody) and F008-009 (an anti-influenza nucleoprotein antibody) were used as negative controls. Anti-FLAG antibody was used for detecting the FLAG tag that had been fused to the recombinant HA. The binding activity is shown as the optical density at 450 nm (OD450).

In order to examine the cross-reactivity to various H5N1 viruses, we prepared recombinant HA molecules from the following seven H5N1 strains: the four kinds of H5N1 vaccine viruses used in the present study (Vie04 (Clade 1), Ind05 (Clade 2.1), Qin05 (Clade 2.2), and Anh05 (Clade 2.3)) and three recently isolated viruses (A/tree sparrow/Indonesia/D10013/2010 (Ind10) (Clade 2.1.3.2a), A/turkey/Egypt/137/2013 (Egy13) (Clade 2.2.1.2), and A/muscovy duck/Vietnam/LBM635/2014 (Vie14) (Clade 2.3.4.4)). The 62 HI-positive clones were subjected to ELISA using these seven different HA molecules. The results are indicated in Figure 3, where the binding to each of the four kinds of vaccine virus particles (Figure 1) was compared to the binding to each of the seven tested HA molecules. 

In most cases, binding specificity was consistent against the particle and the respective purified HA. Among the 62 HI-positive clones, Clone 7-10 showed strong binding to the Qin05 (Clade 2.2) virus particle but weak binding to that of Anh05 (Clade 2.3). On the other hand, Clone 7-10 exhibited binding to all of the tested recombinant H5N1 HA proteins, including those from the three recently isolated viruses. Other than Clone 7-10, only a small number of clones (20-8, 8-1, 4-1, 8-8, 8-9, 20-12, and 11-3) showed binding (albeit weak) to the Ind10 (Clade 2.1.3.2a) virus. Among these Ind10 (Clade 2.1.3.2a)-binding isolates, Clone 11-3 showed peculiar characteristics, as follows. Against virus particles, the 11-3 Ab showed strong binding to Qin05 (Clade 2.2) and weak binding to the other viruses, including the H1N1 and H3N2 viruses. Against the recombinant HA molecules, the 11-3 Ab bound to four strains (Ind05 (Clade 2.1), Qin05 (Clade 2.2), Anh05 (Clade 2.3) and Ind10 (Clade 2.1.3.2a)). Another two clones (13-1 and 14-4) did not show binding to tested HA proteins, although these clones showed HI and neutralizing activities against H5N1 strains (Table 2 and Figure 2). These data appeared to be inconsistent; however, they were obtained with reproducibility. Thus, we concluded that these two clones should have binding activity. It is notable that the two clones bind to virus particle as shown in the Figure 1; however, under the experimental environment, the epitope against these two clones might not be expressed with precise conformations because of local instability on the immunoplate. Thus, 54 of 62 HI-positive clones (87.1%) clones did not show binding to the HAs of recently isolated H5N1 viruses.

### 2.4. Analyses of HI-Negative Clones

#### 2.4.1. Binding Activity to H5N1 Vaccine Strains by the HI-Negative Clones

**Figure 4:**
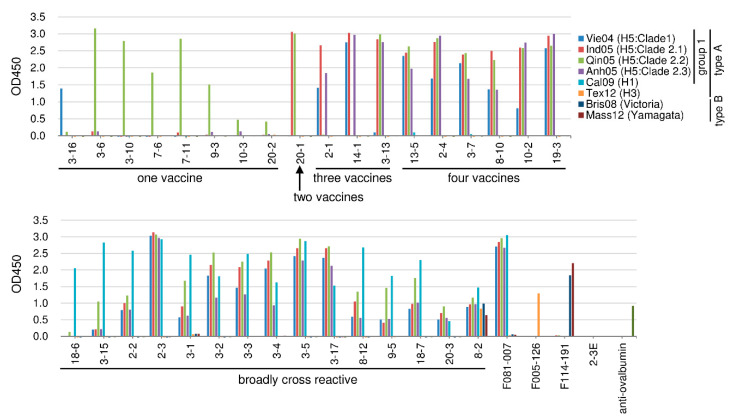
Binding activity of HI-negative clones. Binding activities of thirty-three clones to four H5N1 vaccine strains, H1N1, H3N2, and two kinds of type-B viruses, were examined by ELISA. F081-007 (an anti-H1 and -H5 HA antibody), F005-126 (an anti-H3 HA antibody), and F114-191 (an anti-influenza type-B antibody) were used as positive controls, while 2-3E (anti-rotavirus antibody) was used as a negative control. The binding activity is shown as the optical density at 450 nm (OD450).

The 33 HI-negative clones were subjected to analysis of strain specificity by ELISA as performed above, testing for the binding activity to the four kinds of H5N1 vaccine strain virus particles and to those of H1N1 and H3N2 vaccines, as well as to two type-B vaccines. The results are shown in Figure 4. A total of 15/33 (45%) of the HI-negative clones bound not only to H5N1 viruses but also to H1N1 virus. Most of these H1N1-binding clones also bound to all of the tested H5N1 viruses. Strikingly, Clone 8-2 bound to all of the tested influenza virus particles, including the type-B viruses. The clones that did not bind to H1N1 virus could be divided into four groups based on the number of H5N1 viruses that were bound. Members of the third group (consisting of six clones) bound to four H5N1 viruses; of the fourth group (three clones), to three H5N1 viruses; of the fifth group (one clone), to two H5N1 viruses; and of the sixth group (eight clones), to single H5N1 viruses. Thus, half of the HI-negative Abs showed binding to the H1N1 virus.

#### 2.4.2. H5N1 Virus-Neutralizing Activity among the HI-Negative Clones

**Figure 5:**
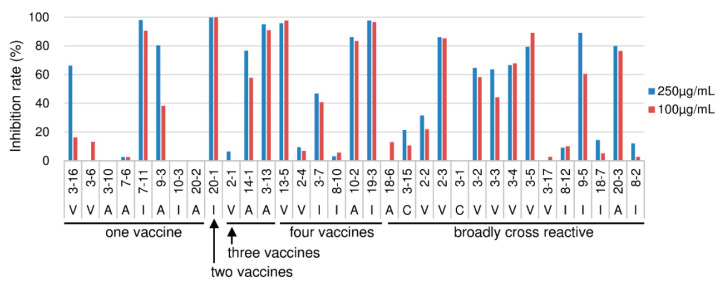
Neutralization activity of Fab-PP from HI-negative clones was measured by focus reduction assay. Neutralization activity was tested at two antibody concentrations (250 µg/mL, blue bars; and 100 µg/mL, red bars). Three kinds of H5N1 viruses were tested, including V: Vie04 (Clade 1), I: Ind05 (Clade 2.1), and A: Anh05 (Clade 2.3); the strain used for each assay is indicated under the clone name. The neutralization activity is shown as the focus reduction rate (%) normalized to that observed in the absence of antibody.

The virus-neutralizing activity of the HI-negative Abs was measured by the focus reduction assay. The results are shown in Figure 5. Although the activities of the clones were widely distributed, a total of 27/33 (81%) of the HI-negative Abs that bound to the target H5N1 virus particles showed some degree of virus-neutralizing activity. The only exceptions were the four isolates represented by clones 3-1, 3-10, 10-3, and 20-2. Thus, most of the HI-negative Abs showed virus-neutralizing activity.

#### 2.4.3. Cross-Reactivity against Recombinant HA Molecule among the HI-Negative Clones

**Figure 6:**
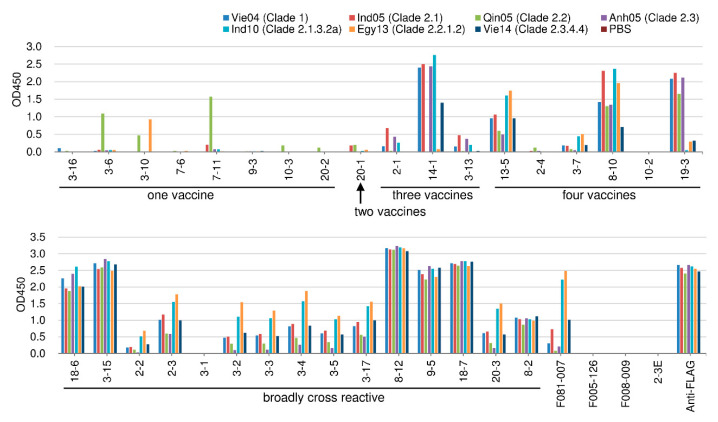
Binding activity of HI-negative clones to recombinant HA molecules derived from seven kinds of H5N1 viruses. Binding was measured by ELISA. F081-007 is an anti-H1 and -H5 HA antibody that was used as a positive control. F005-126 (an anti-H3 HA antibody) and F008-009 (an anti-influenza nucleoprotein antibody) were used as negative controls. Anti-FLAG antibody was used for detecting the FLAG tag that had been fused to the recombinant HA. The binding activity is shown as the optical density at 450 nm (OD450).

The 33 HI-negative clones were subjected to further analysis of binding activity by assessing binding to recombinant HA of the seven H5N1 strains listed above. The results are shown in Figure 6. Four clones, 7-6, 9-3, 10-2, and 3-1, did not show binding to seven recombinant HAs, although these clones showed binding to Qin05 HA that was artificially expressed on the surface of 293T cells (data not shown). Based on the cross-reactivity to recent H5N1 viruses, 21 clones showed binding to recombinant HAs of the three recently isolated viruses that were tested. 

### 2.5. Summary of Cross-Reactivity of Isolated Clones

**Figure 7:**
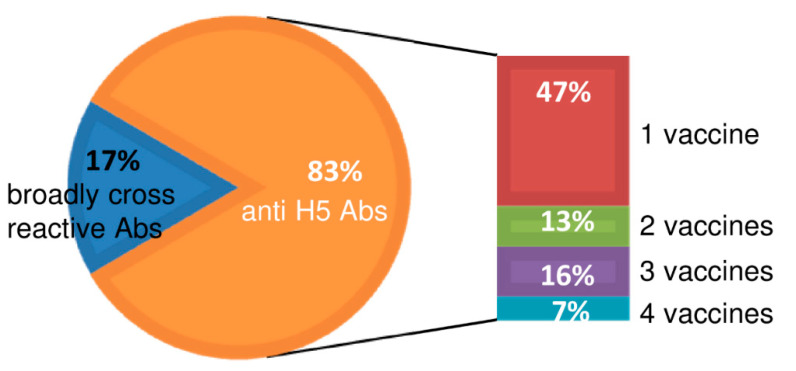
A pie chart indicating the ratio of anti-H5 antibodies that bound only to H5N1 strains compared to broadly cross-reactive antibodies that bound to H5N1, H1N1, H3N2, and type-B strains. Each of the percentages indicates distribution of representative clones categorized based on their cross-reactivity among H5N1 and H1N1 strains. The bar graph shows the percentage of anti-H5 antibodies categorized into 4 groups based on their cross-reactivity among the four H5N1 vaccine strains used in the present study.

The cross-reactivity of representative clones to subtype H1, H3, and type-B viruses (beyond reactivity to strains of H5N1) is summarized in Figure 7. Eighty-three percent of the clones showed H5-specific binding activity, and 17% of the clones showed broad cross-reactivity beyond H5, additionally encompassing H1, H3, H5, and type-B subtypes. Among the clones, 47% showed single specificity, 13% showed dual specificity, 16% showed triple specificity, and 7% showed cross-reactivity against all four of the four tested H5 vaccine virus strains. Although the binding strengths of the clones were widely distributed, all of the clones belonging to the “broadly cross-reactive” class (the 17% noted above) exhibited multiple specificity, with activity against influenza viruses of the H1, H3, H5, and type-B subtypes.

### 2.6. Sequence-Based Analysis of Frequency of Mutation in V_H_-Encoding Gene

Table 3 presents the degree of identity (to germline gene-encoded proteins) of amino acid sequences in the V_H_-encoding gene between each clone and the HI-positive clones (A) and between each clone and that of the HI-negative clones (B). In the case of the HI-positive isolates, most of the clones showed high identity (exceeding 90%), except for two clones, 18-3 (89.8% identity) and 20-12 (85.2%). On the other hand, in the case of the HI-negative isolates, the clones appeared to sort into two groups as follows: lower than 90% identity and higher than 90% identity (to germline gene-encoded proteins). Fourteen of the clones (2-2, 2-3, 3-1, 3-2, 3-3, 3-4, 3-5, 3-7, 3-15, 3-17, 9-5, 18-6, 18-7, and 20-3) showed identities ranging from 72.4 to 88.6%. Thirteen of these 14 isolates (excepting clones 3-7) showed binding to H1N1 virus particles (Figure 4). 

Eighteen clones among the 33 HI-negative clones showed high (>90%) amino acid identity, in the predicted V_H_ domain, to the germline gene-encoded proteins. Among these 18 clones, four (Clones 8-10, 13-5, 14-1, and 19-3) showed broad cross-reactivity to HAs of multiple H5N1 strains (Figure 6). Clones 8-10 and 13-5 exhibited strong binding to the HA proteins of seven H5N1 viruses. Clone 14-1 bound strongly to the HAs of Clade-1, -2.1, -2.3, -2.1.3.2a, and -2.3.4.4 viruses but did not bind to the HAs of Clade-2.2 and Clade-2.2.1.2 viruses (Figure 6), suggesting presence of a shared epitope among H5N1 viruses belonging to clades other than Clade 2.2. Clone 19-3, which bound to four kinds of H5N1 viruses, showed weak binding to the HAs of Egy13 (Clade 2.2.1.2) and Vie14 (Clade 2.3.4.4). Among the isolates that bound to H1N1 virus, only Clone 8-2 had amino acid identity exceeding 90% (achieving 93.2%) compared to the germline-encoded protein. The other 13 HI-negative clones with high (>90%) amino acid identity (vs. the germline-encoded protein) did not show broad cross-reactivity against H5N1 viruses (Figure 6). Thus, most of the HI-negative clones encoding Abs with low mutation frequencies did not show broad cross-reactivity.

### 2.7. Mutation Ratio-Based Evaluation of Cross-Reactive Clones

**Figure 8:**
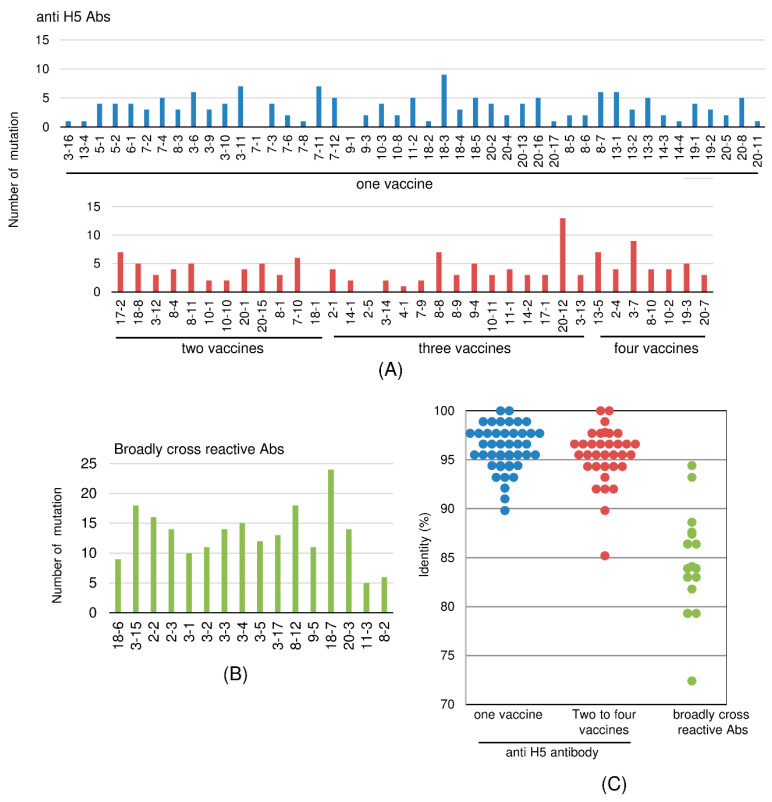
Mutation frequency of amino acid residues in V_H_-encoding region when comparing between the representative clone and the corresponding germline gene. (**A**,**B**) The number of changed amino acid residues introduced by nucleic acid mutation in the V_H_-encoding region. The representative clones are classified into three categories based on their cross-reactivity profiles, as follows: (**A**) one vaccine (singular specificity against one H5N1 strain; blue); two, three, and four vaccines (multiple specificity against H5N1 strains; red); (**B**) Broad cross-reactivity against H1, H3, H5, and type-B viruses; green; (**C**) The degree of identity of amino acid residues in the protein encoded by the V_H_ region of each clone compared to that encoded by the respective germline gene. The identity (%) of each clone shown in Table 3 is presented in a dot plot format for each category described in (**A**). Blue: singular specificity against one H5N1 strain; red: multiple specificity against H5N1 strains; and green: broad cross-reactivity against H1, H3, H5, and type-B viruses.

The identity of various clones to germline-encoded proteins was analyzed and compared with their cross-reactivity. These results (as assessed using the representative clones) are summarized in Figure 8 and Table 3. The clones were classified into three categories according to the range of cross-reactivity: singular specificity (against one H5N1 strain), multiple specificity (against more than one H5N1 strain), and broad cross-reactivity (to H1, H3, H5, and type-B viruses). Most of the wide-range, broadly cross-reactive clones appeared to encode more highly mutated Abs (with greater numbers of amino acid substitutions). Specifically, clones with singular specificity had a mean of 3.4 mutations each and a median of three mutations, and the number of mutations ranged from 0 (0% of the residues) to 9 (10.2% or the residues) at the amino acid level. Clones with multiple specificities against H5N1 strains had a mean of four mutations each and a median of four mutations, and the number of mutations ranged from 0 (0%) mutations to 13 (14.8%) mutations. In contrast, broadly cross-reactive clones had a mean of 13.5 mutations and a median of 13.1 mutations, and the number of mutations ranged from 5 (5.6%) mutations to 24 (27.6%) mutations. 

## 3. Discussion

In the present study, we analyzed the strain specificity of Abs that were present in the blood of volunteers following immunization with four kinds of H5N1 virus that have been stocked as pre-pandemic vaccines to be used for future H5N1 virus pandemics. The isolated Abs were divided into two groups (positive and negative) based on their HI activity. 

The Abs isolated in this study may have been derived from two different types of B cells present at the time of vaccination. The first type of cells would have been naïve B cells that were responding to a first exposure to H5N1 virus (specifically, the virus used as the vaccine). The second type of cells would have been B cells which had been exposed previously to some influenza virus, either by infection or vaccination; this class of B cells would have responded to the present vaccination because the Abs produced by this class of B cells possessed cross-reactivity to the H5N1 viruses used in the present vaccination. Based on this model, we predicted the following differences in Ag specificity between these two types of Ab-producing cells. The first class of cells should produce Abs that bind primarily to H5N1 viruses, while the second class of cells should produce Abs that are able to bind not only to H5N1 virus but also to viruses to which the donor had been previously exposed (e.g., H1N1 vaccine virus). Furthermore, we would expect differences in the mutation frequency in the V_H_-encoding genes in these two classes of B cells: the first class of cells would be expected to harbor genes with relatively lower rates of mutation, while the second class would be expected to harbor genes with relatively higher mutation frequencies. As shown in Figure 8, in most cases, those clones whose mutation frequency was less than 10%, especially those with mutation frequencies close to 0%, were derived from B cells newly induced by vaccination. However, 10% should not be considered to be an absolute threshold for such a classification distinguishing Abs derived from naïve cells from those derived from memory cells. There may exist clones whose mutation frequency deviated from this rule.

Among the HI-positive clones, all represented Abs that were newly induced by vaccination. Notably, most of these clones did not show cross-reactivity to recently isolated H5N1 viruses. Although two clones (7-10 and 11-3) showed broad cross-reactivity, their binding activity varied against different H5N1 viruses. While Clone 7-10 bound to the recombinant HAs of seven H5N1 viruses with equal affinity, large differences were observed in the strength of binding to virus particles of various H5N1 viruses. Thus, we do not expect that Clone 7-10-like Abs would play an important role in counteracting infection by a pandemic-causing H5N1 virus. Among the HI-negative clones, approximately half of these isolates corresponded to B cells (and Abs) that appear to have been present in the blood prior to the present vaccination; the remaining HI-negative clones appeared to correspond to B cells that were newly induced by the present vaccination. Furthermore, the Abs encoded by HI-negative clones corresponding to pre-existing B cells showed broad cross-reactivity, while the Abs encoded by newly induced HI-negative clones exhibited restricted cross-reactivity. These results can be explained as follows. In the case of memory B cells whose growth was stimulated by the present vaccination with H5N1 viruses, such cells presumably were activated during previous exposure to some virus that was different from, but immunologically similar to, the H5N1 vaccine viruses, most likely an H1N1 virus. In the case of Abs produced by such cells, it is likely that the recognized epitopes are shared between, and present on, both H5N1 and H1N1 viruses. Therefore, we would expect such Abs to show broad cross-reactivity against H5N1 viruses. Indeed, among the distinct class of newly induced Abs, only two clones (8-2 and 8-10) showed broad cross-reactivity, binding to the HA of all tested H5N1 viruses.

While Clone 8-2 bound to H1N1 viruses, Clone 8-10 did not. This observation suggests that there exists an HA epitope shared among H5N1 viruses that is distinct from epitopes shared between H1N1 and H5N1 viruses. However, the strength of immunogenicity of this epitope may be relatively low, given that we isolated only one such clone in the present experiment. Since most of the newly induced HI-negative Abs also did not show broad cross-reactivity among H5N1 viruses, we conclude that the present stock of H5N1 vaccine viruses may not work efficiently for the prevention of pandemics caused by H5N1 viruses. 

As shown in Figure 6, the seventeen HI-negative clones (2-2, 2-3, 3-2, 3-3, 3-4, 3-5, 3-7, 3-15, 3-17, 8-2, 8-10, 8-12, 9-5, 13-5, 18-6, 18-7, and 20-3) showed binding to the recombinant HAs of all tested H5N1 viruses. In order to obtain information regarding the genes encoding these Abs and (albeit indirectly) the corresponding epitopes, we compared the following data: (1) the DNA sequences of the germline V_H_- and V_L_-encoding genes carried by each clone, and (2) the amino acid sequence and length of the CDR3 domains of the predicted V_H_ regions (S1 Table). Based on these data, the following characteristics were noted. Eight clones (2-2, 2-3, 3-2, 3-3, 3-4 3-5, 3-7, and 20-3) carried the V_H_1-69 gene (as defined in previous literature), in which the fourth residue of the CDR3 domain was (without exception) predicted to be a tyrosine, consistent with previous literature observations [8,9]. The V_L_ genes carried by these clones were heterogeneous, indicating that the L chain is not directly involved in binding to HA. Two clones (3-15 and 18-7) that strongly bound to the HAs of all tested H5N1 viruses utilized the germline 4-59 sequence as a V_H_-encoding gene and the germline V_κ_1-39 as a V_L_-encoding gene. The predicted CDR3 domains of these two clones were identical to each other except for two residues as follows: DDTGISR(L/V)NAFD(V/I). In the case of these two clones, the encoded Abs strongly bound to H1N1 viruses and the predicted V_H_ protein sequences exhibited 79.3 and 72.4% amino acid identity to the germline gene-encoded protein, suggesting that these clones were derived from memory B cells. Two clones (8-10 and 9-5) that also strongly bound to the HAs of all H5N1 viruses carried the germline 4-4 sequence as the V_H_-encoding gene and the germline V_κ_1-39 sequence as the V_L_-encoding gene. However, Clone 8-10 did not bind to H1N1 virus, while Clone 9-5 did. Furthermore, the identities of amino acid residues in the V_H_ protein (vs. the germline gene-encoded protein) were 95.5 and 87.4%, respectively. These results suggested that Clone 8-10 was newly induced by vaccination, while Clone 9-5 was encoded by a memory B cell. Thus, other than the V_H_1-69 gene, a combination of V_H_4-59 and V_κ_1-39 genes as well as a combination of V_H_4-4 and V_κ_1-39 genes can encode broadly cross-reactive Abs. Clone 8-2 exhibited similar levels of binding to eight kinds of influenza viruses, including H1N1, H3N2, and two types of B viruses (as indicated in Figure 4); this clone also exhibited similar affinity for the HAs of seven kinds of H5N1 viruses (Figure 6). Furthermore, Clone 8-2 showed weak neutralizing activity. This Ab was encoded by V_H_3-7 and V_λ_1-51 genes. These results indicate the presence of a common epitope on the HAs of all influenza viruses. Judging from the degree of identity between germline and somatic V_H_ genes, Clone 8-2 may have been newly induced by the present vaccination. The donor whose blood carried the Ab of Clone 8-2 also produced another highly cross-reactive Ab (Clone 8-10) that did not bind to H1N1 virus but did strongly bind to the HAs of seven H5N1 viruses. Since both Abs (8-2 and 8-10) appear to have been newly induced by the present vaccination (as judged by the degrees of identity to the germline gene-encoded proteins, 93.2 and 94.4%, respectively), the epitopes recognized by these Abs presumably are immunogenic. This person (Donor 8) may possess a special capability for generating highly cross-reactive Ab-producing cells.

**Figure 9:**
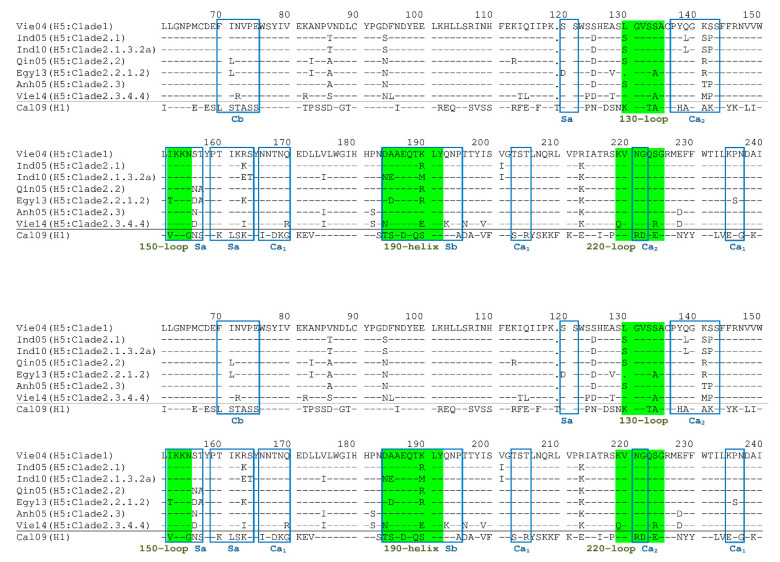
Amino acid sequences of antigenic sites on HAs of H5 and H1 strains used in this study. The sequence of the H5N1 Vie04 strain was used as the standard for comparison. The bars indicate identity to the amino acid in the standard. Amino acid sequences in sites Ca1, Ca2, Cb, Sa, and Sb are boxed in blue. Amino acid sequences in the 130-loop, 150-loop, 190-helix, and 220-loop are shaded in yellow-green. Accession numbers of amino acid sequences: EF541402 (Vie04); CY116646 (Ind05); DQ137873 (Qin05); DQ371928 (Anh05); AB621352 (Ind10); KJ522737 (Egy13); AB979487 (Vie14); FJ966974 (Cal09).

The amino acid sequences of the HA molecules of the seven H5N1 viruses and that of the H1N1 virus used in the present study are shown in Figure 9 [10]. The receptor-binding pocket on this HA molecule is composed of four regions, designated the 130-loop, 150-loop, 190-helix, and 220-loop [11,12]. These regions correspond to amino acid residues 130-135, 152-155, 184-192, and 219-225 (based on H1 amino acid numbering as previously reported in [13]) in the sequences shown in Figure 9. Virus particles bind to target host cells via interaction of the receptor-binding pocket with sialic acid, the receptor on the host cell surface. HI-positive Abs physically block this interaction by binding to the surrounding region of the sialic acid-binding pocket on the HA protein. When many humans have acquired the ability to produce such Abs against the prevalent viruses, a new type of virus starts to appear; this new variant replaces the epitope (amino acids) targeted by the Abs encoded by a HI-negative clone and becomes dominant. The positions of amino acids that have been frequently mutated are restricted and distributed across specific regions. These residues are located in five regions (Sa, Sb, Ca1, Ca2, and Cb), corresponding together to a total of approximately 50 amino acid residues (in the case of H1 virus) [14,15]. The H5 virus belongs to the same group 1 as H1 virus. We compared the differences found in these regions among the HAs of the seven tested H5N1 viruses. Although few differences were observed between Clade-2.1 and -2.1.3.2a viruses or between Clade-2.2 and -2.2.1.2 viruses, very few of the HI-positive Abs showed cross-reactivity against these pairs of virus clades. This result might be explained as follows. The amino acids that differed between the above two pairs of clades presumably are located at sites that serve as targets for HI-positive Abs. These regions correspond to the most immunogenic sites as well as the most readily changed sites (as described above). This hypothesis may explain why the HI-positive Abs newly induced by vaccination with H5N1 viruses in the present study generally do not show broad cross-reactivity.

Next, we consider why many of the memory cells induced by vaccination in this experiment produce broadly cross-reactive Abs. The reason appears to be clear. In this experiment, we performed vaccination with H5N1 viruses. Since all of the volunteers who took part in this project should have been naïve to H5N1 viruses (given that H5N1 viruses normally exist in birds and not in humans), these individuals should not have possessed memory B cells that produced Abs with specificity exclusive to the H5N1 vaccine virus. These memory cells presumably were selected by previous infection by some other viruses (e.g., H1N1). Furthermore, Abs produced by such memory B cells would bind to a shared epitope present on both the H5N1 vaccine virus and the virus that had served as the original immunogen. Thus, these memory B cells were present and able to respond to vaccination with H5N1 viruses. If this explanation is correct, we could argue that this approach is a generally applicable method for inducing B cells that produce broadly cross-reactive Abs. Repetition of natural infection with the same kind of, but immunologically distinct, viruses is expected to result in the appearance of memory B cells producing broadly reacting Abs as follows. First of all, we have to assume the presence of common epitope(s) among the target viruses. Persons who are naive to the target virus will start to produce Abs that can bind to the infecting virus particles in the initial infection. The kind of Abs produced by infection will be determined by the following two factors: (1) the repertoire of Abs formed by naive Ab-producing cells in their body, and (2) the relative immunogenicity of the respective epitopes on the virus particle. For persons who have already experienced infection by immunologically distinct but similar kinds of virus, the response pattern will be classified into two patterns according to the type of epitope and the patient’s memory B cells. If there is no common epitope on the viruses, the response should be the same as that observed upon first infection. On the other hand, under conditions where a shared epitope is present on the viruses, memory B cells producing Abs that can bind to the common epitope are stimulated and amplified. Thus, repetition of natural infection with immunologically distinct viruses of the same kind is expected to induce production of broadly cross-reactive Abs. This scenario explains the process whereby a person gains immuno-protection from influenza viruses.

In the present study, we reported the isolation of several neutralizing Abs showing various levels of cross-reactivity. Many broadly cross-reactive anti-influenza Abs have been reported previously [16,17,18,19,20,21,22,23,24], and the induction of broadly cross-reactive Abs by either infection or vaccination with H5N1 virus or H7N9 virus has been described by several groups [25,26,27,28,29]. Furthermore, we isolated two types of broadly cross-reactive Abs (encoded by Clones 8-10 and 9-5 as well as by Clones 3-15 and 18-7) that are encoded by the same combinations of V_H_- and V_L_-encoding genes. Since these Abs were generated independently in two volunteers, these results suggest that the repertoire of paratopes formed by sets of combinations between germline V_H_- and V_L_-encoding genes in the human body may cover many of the epitopes shared among influenza viruses [30]. However, since most of the newly induced HI-negative Abs did not show broad cross-reactivity among H5N1 viruses, the present stock of H5N1 vaccine viruses may not work efficiently for the prevention of pandemics caused by H5N1 viruses.

## 4. Materials and Methods

### 4.1. Viruses

The following H5N1 viruses were used as precipitated/inactivated vaccines: Clade 1, Vie04 (Kitasato Daiichi Sankyo Company, Limited, Saitama, Japan); Clade 2.1, Ind05 (The Chemo-Sero-Therapeutic Research Institute, Kumamoto, Japan); Clade 2.2, Qin05 (Research Institute for Microbial Diseases, Kagawa, Japan); Clade 2.3, Anh05 (DENKA SEIKEN Co., Ltd., Tokyo, Japan). These four vaccines (before addition of adjuvant) also were used as antigens (Ags) in the screening of Ab libraries, enzyme-linked immunosorbent assay (ELISA), and hemagglutinin inhibition (HI) tests. Four kinds of HA split vaccines (H1N1: A/California/7/2009 (Cal09); H3N2: A/Texas/50/2012 (Tex12); B, Victoria: B/Brisbane/60/2008 (Bris08); B, Yamagata: B/Massachusetts/2/2012 (Mass12)) were used as Ags in ELISA. These split vaccines had been stocked at the Research Institute for Microbial Diseases, Osaka University. The following three live H5N1 viruses were used in the measurement of virus neutralizing activity: Vie04, Ind05, and Anh05.

### 4.2. Ethics Statement

This research was approved by the Ethics Review Committee of Fujita Health University (15-089, 19th May 2015). Written informed consent was obtained from each of the donors.

### 4.3. Construction of Ab-Encoding Libraries

Ab-encoding phage libraries were constructed as described previously [31,32]. Briefly, peripheral blood (100 mL) was collected from each volunteer. Mononuclear cells containing B lymphocytes were isolated from the blood of each donor by using Ficoll, and total RNA was prepared from the cells using ISOGEN (Nippongene, Tokyo, Japan). After synthesis of cDNA from total RNA using Superscript IV (Thermo Fisher Scientific, Tokyo, Japan), DNAs encoding Ab heavy (V_H_-encoding region) chains and light (V_L_-C_L_-encoding region) chains were amplified by PCR. Primers were as described in previous studies [31,32]. After separate construction of libraries encoding heavy (H) and light (L) chains, an Ab-encoding library was constructed by inserting the resulting DNA fragments (corresponding to the V_H_-encoding genes) into the L chain-encoding library as in-frame fusions. 

### 4.4. Screening of the Libraries 

For isolation of clones encoding Abs able to bind to H5N1 virus particles, phage Ab libraries were screened against the four kinds of H5N1 virus particles prepared as vaccines. The screenings were performed by three rounds of panning as follows [31,32]. A Maxisorp tube coated with H5N1 virus was incubated with each phage Ab library and phages bound to the tube were eluted. The eluted phages were infected into *Escherichia coli* DH12S with helper phages and prepared as phages for the next round of panning. After three or four rounds of panning, DH12S infected with the eluted phages were plated onto Luria–Bertani (LB) agar plates containing 100 µg/mL ampicillin and 0.2% glucose without helper phages. Each colony was picked and cultured in yeast extract tryptone (2× YT) medium containing 100 µg/mL ampicillin and 0.05% glucose. The resulting spent medium was expected to contain the Fab-cp3 form of the Ab (i.e., the antigen-binding fragment (Fab) fused with the M13 phage coat protein gp3) by addition of 100 mM isopropyl-β-d-thiogalactopyranoside (IPTG) [33]. The spent medium was cleared by centrifugation at 4 °C, 10,000× *g*, for 10 min, and the resulting supernatant containing the Fab-cp3 Ab was used for ELISA; clones that bound to H5N1 virus were selected for further characterization.

### 4.5. Sequence Analysis 

The nucleotide sequences of the V_H_-encoding fragments of the isolated Ab clones were determined (DNA Sequence Service, Tokyo, Japan; Eurofins Genomics K.K.). The T7ETZ (5′-TAATACGACTCACTATAGGG-3′) oligonucleotide was used as the V_H_ sequencing primer. The methods used to compare Ab-encoding sequences with germline genes were as previously described [34].

### 4.6. Preparation of Fab-PP

Phagemid DNA of each clone encoding a Fab-cp3 of interest was cleaved with SalI to remove the gene encoding cp3 and self-ligated to generate a construct encoding a Fab-PP in which a single domain of protein A (which binds to the Fc portion of IgG) is fused to a Fab fragment [35,36]. After transformation of *E. coli* with the DNA of a Fab-PP-encoding phagemid, secretion of Fab-PP molecules into the culture medium was induced by the addition of IPTG [35]; the Fab-PP was purified from the supernatant of the *E. coli* culture by chromatography with IgG sepharose 6 Fast Flow (Cytiva, Tokyo, Japan).

### 4.7. HI Assay

The HI test was performed as described previously [8]. In brief, purified Fab-PP was adjusted to an initial concentration of 200 µg/mL and subjected to serial dilution in phosphate-buffered saline (PBS) using a 96-well plate. These serial dilutions were preincubated with 4 HA units of virus per well. Guinea pig red cells (0.875% in PBS) were added to each well, and the plate was incubated at room temperature for 60 min. The results are shown as the lowest concentration (in µg/mL) of a given Fab-PP Ab that inhibited hemagglutination.

### 4.8. ELISA

Maxisorp immunoplates were coated with influenza vaccines or recombinant HA (5 µg/mL). After blocking, the wells were incubated with purified Fab-PP Ab. After washing, the wells were further incubated with horse radish peroxidase (HRP)-conjugated anti-human IgG (Fab) (MBL) or anti sheep-HRP Ab (ROCKLAND) as the secondary Ab. The enzyme reactions were initiated by addition of 3,3’,5,5’-tetramethylbenzidine (TMB; Thermo Fisher Scientific) and quenched by addition of H_2_SO_4_. The optical density at 450 nm (OD450) was measured. All experiments were performed in duplicate; the OD450s shown represent the mean of 2 values.

### 4.9. Virus-Neutralizing Activity

For measurement of virus-neutralizing activity, a focus reduction assay was performed by using the single cycling (VN) method [7,37]. An aliquot (250 or 100 µg/mL of Fab-PP Ab) was combined with 100 focus-forming units (FFU) of influenza virus. The resulting mixture was added to each well of a 96-well plate containing Madin–Darby canine kidney (MDCK) cells at 100% confluence, and the plates were incubated at 37 °C for 1 h. After incubation, the cells were washed with serum-free modified Eagle’s medium (MEM) and cultured in MEM supplemented with 0.4% bovine serum albumin (BSA) at 37 °C for 15 h. After incubation, an equal volume of MEM supplemented with 0.4% BSA, 5 µg/mL acetylated trypsin, and 0.5% methyl cellulose was added to each well and the cells were incubated at 37 °C for 28 h. The cells then were fixed with ethanol and stained with peroxidase and anti-peroxidase (PAP). The number of foci was counted. The results are presented as the foci reduction rate (%).

### 4.10. Expression and Purification of Soluble HAs Using Baculovirus Expression Systems

Soluble HAs were prepared by utilizing the Bac-to-Bac Baculovirus Expression Systems using pFastBac1 (Thermo Fisher Scientific, Inc.). Mature HA ectodomain from A/bar-headed goose/Qinghai/0510/05(H5N1) (accession number: DQ137873) that was fused with peptides, as described below, was artificially synthesized (GeneArt Gene Synthesis service; Thermo Fisher Scientific, Inc.). The resulting fragment was cloned between the BamHI and HindIII sites of pFastBac1 to generate a donor plasmid designated pFB-H5Qinghai2005. The mature ectodomain of HA was fused (at the ectodomain’s N-terminus) with a gp64 signal peptide derived from pBAC-3 (Novagen, Tokyo, Japan) at the N-terminal and (at the ectodomain’s C-terminus) with (in order) a thrombin protease cleavage site, a trimerization “foldon” sequence [38], a FLAG tag, and a His-tag. To generate recombinant bacmid, the resultant transformants were inoculated in super optimal broth with catabolites repression (SOC) medium and shaken at 37 °C for 4 h. *E. coli* cells containing the recombinant bacmid were plated on LB agar plates, and the plates were incubated. The recombinant bacmid was isolated by picking white colonies from the plates; the bacmid then was used to transfect Sf9 cells. After culturing of the Sf9 cells, the culture supernatant containing the resultant recombinant baculoviruses was recovered and used for infection of Sf9 cells. Finally, Sf9 cells were infected with the recombinant baculoviruses and cultured in SF-900 II SFM at 27 °C for 72 h; the soluble HA secreted into the culture medium then was purified from the supernatant of the spent medium by chromatography with Ni-NTA Agarose columns (QIAGEN, Tokyo, Japan).

## Figures and Tables

**Table 1:** Summary of the combination of H5N1 vaccines used for vaccination, collection of mononuclear cells, construction of Ab-encoding libraries, and isolation of clones.

Donor	Gender	Age	Vaccination		Number of Mononuclear Cells	Clone Size of Ab Library	Number of Isolated Clones
			First	Second			
1	M	67	Vie04 (1) ^a^	Vie04 (1)	1.86 × 10^8^	2.89 × 10^9^	72
2	M	72	Vie04 (1)	Ind05 (2.1)	1.23 × 10^8^	1.73 × 10^9^	160
3	F	67	Vie04 (1)	Qin05 (2.2)	9.10 × 10^7^	1.12 × 10^9^	176
4	M	40	Vie04 (1)	Anh05 (2.3)	1.23 × 10^8^	1.96 × 10^9^	133
5	M	54	Ind05 (2.1)	Vie04 (1)	1.61 × 10^8^	1.30 × 10^9^	149
6	F	44	Ind05 (2.1)	Ind05 (2.1)	3.30 × 10^8^	1.00 × 10^9^	65
7	M	29	Ind05 (2.1)	Qin05 (2.2)	1.50 × 10^8^	5.48 × 10^9^	167
8	F	28	Ind05 (2.1)	Anh05 (2.3)	9.94 × 10^7^	1.03 × 10^9^	156
9	M	56	Qin05 (2.2)	Vie04 (1)	1.75 × 10^8^	2.22 × 10^9^	159
10	M	49	Qin05 (2.2)	Ind05 (2.1)	8.19 × 10^7^	4.76 × 10^9^	174
11	M	51	Qin05 (2.2)	Qin05 (2.2)	1.73 × 10^8^	2.02 × 10^9^	61
13	M	31	Anh05 (2.3)	Vie04 (1)	1.26 × 10^8^	2.88 × 10^9^	160
14	F	60	Anh05 (2.3)	Ind05 (2.1)	8.67 × 10^7^	4.33 × 10^9^	137
16	M	33	Anh05 (2.3)	Anh05 (2.3)	1.37 × 10^8^	1.66 × 10^9^	3
17	M	40	Vie04 (1)	Ind05 (2.1)	1.44 × 10^8^	2.01 × 10^9^	144
18	M	41	Vie04 (1)	Qin05 (2.2)	6.42 × 10^7^	6.33 × 10^9^	115
19	M	49	Ind05 (2.1)	Anh05 (2.3)	8.94 × 10^7^	6.72 × 10^9^	200
20	M	40	Qin05 (2.2)	Anh05 (2.3)	2.12 × 10^8^	3.84 × 10^9^	230

^a^ Clade of each strain is shown in parentheses.

**Table 2:** Measurement of HI (hemagglutination inhibition) activity.

Clone	Strain	HI Activity (µg/mL)
2-5	Ind05 (2.1) ^a^	6.25
3-9	Qin05 (2.2)	100
3-11	Qin05 (2.2)	3.13
3-12	Qin05 (2.2)	12.5
3-14	Qin05 (2.2)	25
4-1	Anh05 (2.3)	0.78
5-1	Ind05 (2.1)	6.25
5-2	Ind05 (2.1)	12.5
6-1	Ind05 (2.1)	12.5
7-1	Qin05 (2.2)	6.25
7-2	Ind05 (2.1)	12.5
7-3	Qin05 (2.2)	50
7-4	Ind05 (2.1)	50
7-8	Ind05 (2.1)	50
7-9	Ind05 (2.1)	50
7-10	Qin05 (2.2)	100
7-12	Qin05 (2.2)	6.25
8-1	Anh05 (2.3)	25
8-3	Ind05 (2.1)	6.25
8-4	Ind05 (2.1)	12.5
8-5	Anh05 (2.3)	12.5
8-6	Anh05 (2.3)	12.5
8-7	Anh05 (2.3)	12.5
8-8	Ind05 (2.1)	6.25
8-9	Ind05 (2.1)	3.13
8-11	Ind05 (2.1)	12.5
9-1	Qin05 (2.2)	50
9-4	Qin05 (2.2)	6.25
10-1	Qin05 (2.2)	25
10-8	Qin05 (2.2)	50
10-10	Qin05 (2.2)	25
10-11	Qin05 (2.2)	12.5
11-1	Qin05 (2.2)	6.25
11-2	Qin05 (2.2)	12.5
11-3	Qin05 (2.2)	6.25
13-1	Anh05 (2.3)	50
13-2	Anh05 (2.3)	3.13
13-3	Anh05 (2.3)	6.25
13-4	Vie04 (1)	12.5
14-2	Ind05 (2.1)	3.13
14-3	Anh05 (2.3)	25
14-4	Anh05 (2.3)	12.5
17-1	Ind05 (2.1)	1.56
17-2	Vie04 (1)	6.25
18-1	Qin05 (2.2)	25
18-2	Qin05 (2.2)	25
18-3	Qin05 (2.2)	25
18-4	Qin05 (2.2)	100
18-5	Qin05 (2.2)	25
18-8	Qin05 (2.2)	12.5
19-1	Anh05 (2.3)	6.25
19-2	Anh05 (2.3)	12.5
20-4	Qin05 (2.2)	12.5
20-5	Anh05 (2.3)	12.5
20-7	Qin05 (2.2)	100
20-8	Anh05 (2.3)	6.25
20-11	Anh05 (2.3)	6.25
20-12	Qin05 (2.2)	3.13
20-13	Qin05 (2.2)	25
20-15	Qin05 (2.2)	50
20-16	Qin05 (2.2)	12.5
20-17	Qin05 (2.2)	50

^a^ The clade of each strain is shown in parentheses.

**Table:** (**A**)

Clone	Germline	Identity (%)
2-5	4-34*01	100.0
3-9	4-4*02	96.6
3-11	4-31*03	91.0
3-12	4-34*01	96.6
3-14	4-34*01	97.7
4-1	3-9*01	98.9
5-1	3-9*01	95.5
5-2	3-9*01	95.5
6-1	4-34*01	95.4
7-1	3-9*01	100.0
7-2	3-20*01	96.6
7-3	3-21*01	95.5
7-4	3-23*01	94.3
7-8	4-34*01	98.9
7-9	4-34*01	97.7
7-10	4-39*01	93.2
7-12	4-39*01	94.4
8-1	1-18*01	96.6
8-3	3-9*01	96.6
8-4	3-9*01	95.5
8-5	3-9*01	97.7
8-6	3-9*01	97.7
8-7	3-21*01	93.2
8-8	3-33*01	92.0
8-9	3-43*01	96.6
8-11	4-34*01	94.3
9-1	3-9*01	100.0
9-4	3-66*01	94.3
10-1	1-8*01	97.7
10-8	3-33*01	97.7
10-10	4-39*01	97.8
10-11	4-34*01	96.6
11-1	1-18*04	95.5
11-2	4-30-2*01	94.4
11-3	4-31*03	94.4
13-1	1-8*01	93.2
13-2	1-18*01	96.6
13-3	3-9*01	94.3
13-4	3-33*06	98.9
14-2	3-30*04	96.6
14-3	4-34*01	97.7
14-4	4-4*02	98.9
17-1	4-34*01	96.6
17-2	4-59*01	92.0
18-1	3-9*01	100.0
18-2	3-11*01	98.9
18-3	3-33*06	89.8
18-4	4-31*03	96.6
18-5	4-31*03	94.4
18-8	5-51*01	94.3
19-1	3-7*01	95.5
19-2	3-20*01	96.6
20-4	3-9*01	97.7
20-5	3-9*01	97.7
20-7	3-9*01	96.6
20-8	3-9*01	95.5
20-11	3-20*01	97.7
20-12	3-33*01	85.2
20-13	3-53*01	95.4
20-15	4-34*01	94.3
20-16	4-39*01	94.4
20-17	4-59*01	98.9

**Table:** (**B**)

Clone	Germline	Identity (%)
2-1	1-69*01	95.5
2-2	1-69*01	81.8
2-3	1-69*01	83.9
2-4	3-30-3*01	95.5
3-1	1-2*01	88.6
3-2	1-69*01	86.4
3-3	1-69*01	84.1
3-4	1-69*01	83.0
3-5	1-69*01	86.4
3-6	1-69*04	93.2
3-7	1-69*06	85.2
3-10	4-4*02	95.5
3-13	4-34*01	96.6
3-15	4-59*01	79.3
3-16	5-51*01	98.9
3-17	5-51*01	83.0
7-6	4-4*02	97.7
7-11	4-39*01	92.1
8-2	3-7*02	93.2
8-10	4-4*02	95.5
8-12	4-59*01	79.3
9-3	3-30*01	97.7
9-5	4-4*08	87.4
10-2	1-46*03	95.5
10-3	3-9*01	95.5
13-5	4-34*01	92.0
14-1	3-21*01	97.7
18-6	4-39*01	87.6
18-7	4-59*01	72.4
19-3	3-21*01	94.3
20-1	1-2*02	95.5
20-2	1-18*01	95.5
20-3	1-69*01	83.9

## Supplementary Materials

Supplementary materials can be found at https://www.mdpi.com/1422-0067/21/19/7422/s1.

## Abbreviations

Abs	antibodies
HI	hemagglutination inhibition
HPAI	highly pathogenic avian influenza
HA	hemagglutinin
Vie04	A/Vietnam/1194/2004 (Clade 1) (H5N1)
Ind05	A/Indonesia/5/2005 (Clade 2.1) (H5N1)
Qin05	A/Qinghai/1A/2005 (Clade 2.2) (H5N1)
Anh05	A/Anhui/1/2005 (Clade 2.3) (H5N1)
ELISA	enzyme-linked immunosorbent assay
Cal09	A/California/7/2009 (H1N1)
Tex12	A/Texas/50/2012 (H3N2)
Bris08	B/Brisbane/60/2008
Mass12	B/Massachusetts/2/2012
LB	Luria–Bertani
2× YT	yeast extract tryptone
Fab	antigen-binding fragment
IPTG	isopropyl-β-d-thiogalactopyranoside
PBS	phosphate-buffered saline
HRP	horse radish peroxidase
TMB	3,3’,5,5’-tetramethylbenzidine
FFU	focus-forming units
MDCK	Madin–Darby canine kidney
MEM	modified Eagle’s medium
BSA	bovine serum albumin
PAP	peroxidase and anti-peroxidase
SOC	super optimal broth with catabolites repression
X-Gal	5-bromo-4-chloro-3-indolyl-β-d-galactoside
CDR3	complementarity-determining region 3
Egy13	A/turkey/Egypt/137/2013 (Clade 2.2.1.2) (H5N1)
Vie14	A/muscovy duck/Vietnam/LBM635/2014 (Clade 2.3.4.4) (H5N1)
