# Supplementary material for: Comprehensive Analysis of Antibodies Induced by Vaccination with 4 Kinds of Avian Influenza H5N1 Pre-Pandemic Vaccines
Source: Int J Mol Sci. 2020 Oct 8;21(19):7422. doi: 10.3390/ijms21197422 (PMC7582428; doi:10.3390/ijms21197422)
Supplement: Supplementary file 1 [file ijms-21-07422-s001.pdf]

		VH germline Gene				VL germline gene	
Clone	Number of isolated clone	V Gene	D Gene	J Gene	CDR3 VH	V Gene	λ or κ
2-5	45	4-34*01	2-21*02	2*01	ARALEVTAPLYWYFEL	2-14*01	λ
2-1	2	1-69*01	3-10*01	4*02	ARGDLYHTSGSYRGRFDY	2-28*01	κ
2-6	1	1-69*01	3-10*01	4*02	ARGDLYHTSGSYRGRFDY		
2-4	27	3-30-3*01	3-22*01	4*02	ARDLLRDYYDSSGPFDY	2-14*01	λ
2-2	18	1-69*01	3-10*01	6*02	ARGDGYYFGSGSGLDV	1-39*01	κ
2-7	12	1-69*01	3-10*01	6*02	ARGDGYYFGSGSGLDV		
2-8	10	1-69*01	3-10*01	6*02	ARGDGYYFGSGSGLDV		
2-9	8	1-69*01	3-10*01	6*02	ARGDGYYFGSGSGLDV		
2-10	7	1-69*01	3-10*01	6*02	ARGDGYYFGSGSGLDV		
2-13	3	1-69*01	3-10*01	6*02	ARGDGYYFGSGSGLDV		
2-14	3	1-69*01	3-10*01	6*02	ARGDGYYFGSGSGLDV		
2-17	1	1-69*01	3-10*01	6*02	ARGDGYYFGSGSGLDV		
2-18	1	1-69*01	3-10*01	6*02	ARGDGYYFGSGSGLDV		
2-19	1	1-69*01	3-10*01	6*02	ARGDGYYFGSGSGLDV		
2-20	1	1-69*01	3-10*01	6*02	ARGDGYYFGSGSGLDV		
2-11	7	1-69*01	3-10*01	6*02	ARSDGYYFGSGSGLDV		
2-12	4	1-69*01	3-10*01	6*02	ARSDGYYFGSGSGLDV		
2-15	1	1-69*01	3-10*01	6*02	ARSDGYYFGSGSGLDV		
2-16	1	1-69*01	3-10*01	6*02	ARSDGYYFGSGSGLDV		
2-3	1	1-69*01	3-22*01	4*02	AGHPYYYDTSPLSI	1-27*01	κ
3-9	1	4-4*02	3-9*01	4*02	ARDLGYDILTGFYGYFDY	2-14*01	λ
3-11	6	4-31*03	3-9*01	4*02	ARGGLVDILTGYPLDY	1-12*01	κ
3-12	4	4-34*01	2-8*02	6*02	ARVGGGLVDYYYYGMDV	1-40*01	λ
3-14	6	4-34*01	5-24*01	6*02	ARGGTITEYYYYGMDV	1-40*01	λ
3-18	2	4-34*01	5-24*01	6*02	ARGGTITEYYYYGMDV		
3-1	3	1-2*05	6-13*01	6*02	AREVKRDSSSWYNNYYVMDV	1-39*01	κ
3-19	1	1-2*05	6-13*01	6*02	AREVKRDSSSWYNNYYVMDV		
3-20	1	1-2*05	6-13*01	6*02	AREVKRDSSTWYNNYYVMDV		
3-10	1	4-4*02	3-22*01	6*02	ARYSPSFYYYDSSANYYHGMDV	1-39*01	κ
3-13	1	4-34*01	3-9*01	4*02	ARVGPDIFTDYSKYYFDY	2-14*01	λ
3-7	1	1-69*06	2-21*01	4*02	ARSPHIYPPYYFDS	1-5*03	κ
3-16	2	5-51*01	3-9*01	4*02	ARLLRGGTYYDILTGYPELGY	1-44*01	λ
3-22	1	5-51*01	3-9*01	4*02	ARLLRGGTYYDILTGYPELGY		
3-6	65	1-69*04	2-2*01	6*02	ARGAGCTSTSCYVGDMDV	2-28*01	κ
3-15	6	4-59*01	7-27*01	3*01	ARDDTGISRLNAFDV	1-39*01	κ
3-2	1	1-69*01	3-10*01	1*01	AEANDYYGSSHFQN	1-5*01	κ
3-3	1	1-69*01	3-10*01	6*02	ARGTDYYGSSPLDV	1-27*01	κ
3-23	1	1-69*01	3-10*01	6*02	ARGTDYYGSSPLDV		
3-4	4	1-69*01	1-26*01	4*02	ARGRNYYIDYFEY	4-1*01	κ
3-25	1	1-69*01	1-26*01	4*02	ARGRNYYIDYFEY		
3-24	1	1-69*01	3-16*01	4*02	ARGRNYYVDFFES		
3-5	18	1-69*01	3-22*01	4*02	ARSSGYHFTSNYFDY	1-39*01	κ
3-28	1	1-69*01	3-22*01	4*02	ARSSGYHFTSNYFDY		
3-27	1	1-69*01	3-22*01	4*02	ARSSGYHLTSNYFDY		
3-26	1	1-69*01	3-22*01	4*02	ARSSGYHITSNYFDH		
3-17	25	5-51*01	3-9*01	4*02	TSSKPILRYFGWQLPPPFDS	2-14*03	λ
3-29	1	5-51*01	3-9*01	5*01	ASSKPILRYFDWQLPPPFDS		
4-1	51	3-9*01	3-22*01	5*02	AKGDSYDSSGSYWNNWFDP	1-40*01	λ
4-2	30	3-9*01	3-22*01	5*02	AKGDSYDSSGSYWNNWFDP		
4-4	1	3-9*01	3-22*01	5*02	AKGDSYDSSGSYWNNWFDP		
4-3	2	3-9*01	3-22*01	5*02	AKGDSYDSSGYYYRNWFDP		
4-5	3	3-9*01	3-22*01	5*02	AKGESYDSSGHPAVNWFDP		
5-1	22	3-9*01	3-16*01	6*02	AKDIGGPASDYYYYGMDV	2-14*01	λ
5-3	14	3-9*01	3-10*0	6*02	AKDISGPAGDYYYYGMDV		
5-4	10	3-9*01	2-2*01	6*02	AKDIGGPAGDYYYYGMDV		
5-6	2	3-9*01	2-2*01	6*02	AKDIGGPAGDYYYYGMDV		
5-7	1	3-9*01	2-2*01	6*02	AKDIGGPAGDFYYYGMDV		
5-5	3	3-9*01	2-2*01	6*02	AKDIGGPAADYYYYGMDV		
5-2	18	3-9*01	4-23*01	6*02	AKDIGGPTDYYYYGMDV	2-14*01	λ
5-8	13	3-9*01	4-23*01	6*02	AKDIGGPTDYYYYGMDV		
5-10	1	3-9*01	4-23*01	6*02	AKDIGGPTDYYYYGMDV		
5-9	2	3-9*01	4-23*01	6*02	AKDIGGPTDYYYYGLDV		
6-1	40	4-34*01	3-10*01	3*02	ARGPTLTMVRETAFDI	1-44*01	λ
6-2	21	4-34*01	3-10*01	3*02	ARGPTLTMVRETAFDI		
6-3	3	4-34*01	3-10*01	3*02	ARGPTPTMVRDAAFDI		
6-4	1	4-34*01	3-10*01	3*02	ARGPTPTMVRDAAFDI		
7-3	1	3-21*01	5-12*01	3*02	AKTLGRLRFPPEDAFDI	2-14*01	λ
7-13	1	3-21*01	5-12*01	3*02	AKTLGRLRFPPEDAFDI		
7-12	8	4-39*01	3-9*01	4*02	ARRVLDDILTGSGFDY	1-5*01	κ
7-1	1	3-9*01	6-19*01	3*01	AKDYSSGWDHDAFNY	1-40*01	λ
7-2	31	3-20*01	6-13*01	4*02	AREDFLAAARSAPDY	1-47*01	λ
7-15	1	3-20*01	6-13*01	4*02	AREDFLAAARSAPDY		
7-14	14	3-20*01	6-13*01	4*02	ARQDLLAAAGGAPDY		
7-4	10	3-23*01	4-17*01	4*02	AKDLPYGDEHPRFGY	1-39*01	κ
7-16	1	3-23*01	4-17*01	4*02	AKDLPYGDEHPRFGY		
7-8	1	4-34*01	3-10*01	5*02	ARGDTMVREKVWFDP	2-14*01	λ
7-9	33	4-34*01	4-17*01	5*02	ARAGDYLTNVGDNWFDP	1-40*01	λ
7-17	3	4-34*01	4-17*01	5*02	ARAGDYLTNVGDNWFDP		
7-19	1	4-34*01	4-17*01	5*02	ARAGDYLTNVGDNWFDP		
7-20	1	4-34*01	4-17*01	5*02	ARAGDSLTNVGDNWFDP		
7-18	2	4-34*01	4-17*01	5*02	ARDGDFLTNVGDNWFDP		
7-6	2	4-4*02	4-23*01	6*02	ARRGVTPSYYYHGMDV	1-39*01	κ
7-21	1	4-4*02	4-23*01	6*02	ARRGVTPSYYYHGMDV		
7-10	2	4-39*01	3-9*01	4*02	ASGRLSDILTAYYFDY	1-5*03	κ
7-11	45	4-39*01	3-9*01	4*02	ARHPLVDILTAWPFDY	1-5*03	κ
7-22	2	4-39*01	3-9*01	4*02	ARHPLVDILTGWPFDY		
8-5	1	3-9*01	3-22*01	5*02	AKGDAYDSSGYQANWFDP	1-40*01	λ
8-6	2	3-9*01	6-13*01	5*02	AKGDAVAAGHWVMNWFDP	1-40*01	λ
8-7	46	3-21*01	5-18*01	5*02	ARLAYNYGPRAENWFDP	1-44*01	λ
8-1	1	1-18*01	6-19*01	5*02	ARIVGYSSGWYGWFDP	1-40*02	λ
8-3	3	3-9*01	2-2*01	6*02	AKDIGGPAYDYYYYGMDV	2-14*01	λ
8-13	1	3-9*01	2-2*01	6*02	AKDIGGPAYDYYYYGMDV		
8-4	1	3-9*01	2-2*01	6*03	AKDMRILPDTVVNIMDV	2-14*01	λ
8-14	1	3-9*01	2-2*01	6*03	AKDMRILPDTVVNIMDV		
8-11	2	4-34*01	2-21*02	3*02	AKEGLVTEISAFDI	1-51*01	λ
8-15	1	4-34*01	2-21*02	3*02	AREGLVTEINAFDI		
8-9	36	3-43*01	2-2*01	6*02	AKEGGVVPDEGYYYHYGMDV	1-51*01	λ
8-16	1	3-43*01	2-2*01	6*02	AKEGGVVPDEGYYYHYGMDV		
8-17	29	3-43*01	2-2*01	6*02	AKEGGVVPDEGYYYHYGMDV		
8-8	6	3-33*01	6-19*01	6*02	ARDLELPPFSFSSGHSWGMDV	2-14*01	λ
8-18	5	3-33*01	6-19*01	6*02	ARDLELPPFSFSSGHSWGMDV		
8-20	10	3-33*01	6-19*01	6*02	ARDLELPPFSFSSGHSWGMDV		
8-10	3	4-4*02	2-2*01	5*02	ARVTSDCSSAGCSSYNWFDP	1-39*01	κ
8-12	5	4-59*01	7-27*01	3*01	ARDDTGISRLNAFDV	1-39*01	κ
8-2	1	3-7*02	6-13*01	4*02	VRAIGAAGSY	1-51*01	λ
9-1	2	3-9*01	5-24*01	3*02	AKDIYHLLEMATEPYAFDI	1-39*01	κ
9-4	31	3-66*01	5-18*01	3*02	ARGGEVTGWGFGAFDI	3-20*01	κ
9-6	25	3-66*01	5-18*01	3*02	ARGGEVTGWGFGAFDI		
9-3	1	3-30*04	3-22*01	4*02	ARDLLRDYYESTGYHDY	2-23*02	λ
9-5	13	4-59*01	1-14*01	3*02	AREYRYNPQLLSYFDAFDI	1-39*01	κ
9-7	8	4-59*01	1-14*01	3*02	AREYRYNPQLLSYFDAFDI		
10-1	1	1-8*01	5-24*01	6*02	ARGSGDGYYNAEIYYYSGMDV	2-11*01	λ
10-8	2	3-33*01	3-10*01	3*02	ARDMDTPDAFDI	1-40*01	λ
10-10	17	4-39*01	2-2*02	4*02	ARHDCDNTGCYTEVYAHPLDY	1-47*01	λ
10-12	2	4-39*01	2-2*02	4*02	ARHDCDNTGCYTEVYAHPLDY		
10-11	6	4-34*01	1-1*01	5*02	ARGSSATGEYNWFDP	2-14*01	λ
10-14	5	4-34*01	1-1*01	5*02	ARGSSATGEYNWFDP		
10-17	1	4-34*01	1-1*01	5*02	ARGSSATGEYNWFDP		
10-13	6	4-34*01	6-6*01	5*02	ARGSSAVGEYNWFDP		
10-15	3	4-34*01	6-6*01	5*02	ARGSSAVGEYNWFDP		
10-16	1	4-34*01	6-6*01	5*02	ARGSSAVGEYNWFDP		
10-3	3	3-9*01	3-22*01	4*02	AKGVIDGYEHFDY	1-40*01	λ
10-2	92	1-46*01	4-17*01	1*01	ARAGDGDYGSWWYFQH	1-39*01	κ
10-18	11	1-46*01	4-17*01	1*01	ARAGDGDYGSWWYFQH		
10-19	1	1-46*01	4-17*01	1*01	ARAGDGDYGSWWYFQH		
11-2	6	4-30-2*01	4-17*01	3*02	ARVNGDYVEPWMSAFDI	1-47*01	λ
11-4	4	4-30-2*01	4-17*01	3*02	ARVNGDYVEPWMSAFDI		
11-5	1	4-30-2*01	4-17*01	3*02	ARVNGDYVEPWMSAFDI		
11-3	21	4-31*03	3-9*01	5*02	ARGHLQDILTGYWFDP	1-12*01	κ
11-10	1	4-31*03	3-9*01	5*02	ARGHLQDILTGYWFDP		
11-6	5	4-31*03	3-9*01	5*02	ARGTLDDILTGYPLDP		
11-7	2	4-31*03	3-9*01	5*02	ARGTLDDILTGYPLDP		
11-8	1	4-31*03	3-9*01	5*02	ARGTLDDILTGYPLDP		
11-9	1	4-31*03	3-9*01	5*02	ARGTLDDILTGYPLDP		
11-11	1	4-31*03	3-9*01	4*02	ARGGLDDILTGYYFDY		
11-12	1	4-31*03	3-9*01	4*02	ARGGLDDILTGYYFDY		
11-1	5	1-18*04	3-22*01	3*02	ARDLAQSITLITEGSFDI	1-44*01	λ
11-14	3	1-18*04	3-22*01	3*02	ARDLAQSITLITEGSFDI		
11-15	1	1-18*04	3-22*01	3*02	ARDLAQSITLITEGSFDI		
13-4	77	3-33*06	4-17*01	1*01	AKDPAYYGDYVSLGYFQH	1-12*01	κ
13-6	4	3-33*06	4-17*01	1*01	AKDPAYYGDYGSLGYFQH		
13-3	1	3-9*01	3-22*01	5*02	AKGDAYDSSGYQLNWFDP	1-40*01	λ
13-2	30	1-18*01	3-10*01	6*03	ATNYYGSGSDLDV	1-40*01	λ
13-7	17	1-18*01	3-10*01	6*03	ATNYYGSGSDLDV		
13-1	10	1-8*01	3-10*01	6*03	ARVIGYYGSGTYLEGYYYYMDL	1-39*01	κ
13-8	8	1-8*01	3-10*01	6*03	ARVIGYYGSGTYLEGYYYYMDL		
13-9	1	1-8*01	3-10*01	6*03	ARVIGYYGSGTYLEGYYYYMDL		
13-5	7	4-34*01	3-9*01	5*02	ARGNPQLQYFDWQRRPLDP	2-8*01	λ
14-2	27	3-30*04	2-2*01	5*02	ARHACRSTSCPSPVDNWFDP	1-39*01	κ
14-5	10	3-30*04	2-2*01	5*02	ARHACRSTSCPSPVDNWFDP		
14-3	24	4-34*01	2-2*01	3*02	ARINCSSPSCYDAFDI	3-1*01	
14-4	40	4-4*02	3-3*01	6*03	ARGTIFGEYYFYYYMDV	1-40*01	λ
14-6	12	4-4*02	3-3*01	6*03	ARGTIFGEYYFYYYMDV		
14-1	11	3-21*01	3-16*01	6*02	AREGADANYYYYAMDV	1-39*01	κ
14-7	2	3-21*01	3-16*01	6*02	AREGADANYYYYAMDV		
14-8	6	3-21*01	3-16*01	6*02	AREGADANYYYYAMDV		
14-9	1	3-21*01	3-16*01	6*02	AREGADANYYYYAMDV		
17-1	67	4-34*01	4-23*01	5*02	ARAGLMTTVGWFDP	2-14*01	λ
17-2	61	4-59*01	3-10*01	4*02	ARDAPTPYGSGLYFFDY	3-25*01	λ
17-3	5	4-59*01	3-10*01	4*02	ARDAPTPYGSGLYFFDY		
17-4	6	4-59*01	3-10*01	4*02	ARDAPTPYGSGLYFFDY		
17-5	1	4-59*01	3-10*01	4*02	ARDAPTPYGSGLYFFDY		
18-8	25	5-51*01	3-9*01	3*02	ARHGLFDFEPNLGGFDI	1-47*01	λ
18-4	1	4-31*03	3-9*01	4*02	ARNPLDDILTPGYFDY	1-12*01	κ
18-5	2	4-31*03	3-9*01	4*02	ARDTLDDILTPGYFDY	1-12*01	κ
18-2	2	3-11*01	6-19*01	5*02	ARDQWYTSDWTELHWFDP	1-40*01	λ
18-1	2	3-9*01	3-22*01	5*02	AKGDSYDSSGHYWSNWFDP	1-40*01	λ
18-3	14	3-33*06	3-9*01	4*02	AKDLGARDIMTSDVFDY	1-44*01	λ
18-10	3	3-33*06	5-12*01	4*02	AKDLGARDIMPSDVFDY		
18-6	1	4-39*01	2-15*01	3*02	ARLSRTMSLDLRIYAFDI	2-14*01	λ
18-7	18	4-59*01	1-14*01	3*02	VRDDTGISRVNAFDI	1-39*01	κ
19-1	88	3-7*01	3-9*01	4*02	ARADHYDILTGFWGWSDY	1-40*01	λ
19-4	3	3-7*01	3-9*01	4*02	ARADHYDILTGFWGWSDY		
19-2	2	3-20*01	3-10*01	5*02	ARVDHYASGSYEGWFDP	1-40*01	λ
19-3	5	3-21*01	3-10*01	6*02	ARGGIGELFYYYYGMDV	3-19*01	λ
20-5	4	3-9*01	2-2*01	5*02	AKGDNYGSSMSWLNNWFDP	1-40*01	λ
20-11	3	3-20*01	6-19*01	5*02	ARVDAYSTGWYVGWFDP	1-50*01	λ
20-13	2	3-53*01	6-19*01	3*02	ARAESSGWTDAFDI	1-40*01	λ
20-18	2	3-53*01	6-19*01	3*02	ARAESSGWTDAFDI		
20-4	1	3-9*01	2-2*01	4*02	VKDGGTVVVPPDLYSCFDY	1-40*01	λ
20-17	31	4-59*01	3-22*01	5*02	ARGEAYYDTTGYENWFDP	1-44*01	λ
20-15	1	4-34*01	3-16*01	6*02	ARELGQWVEYYYAMDV	2-14*01	λ
20-12	22	3-33*01	5-12*01	4*02	ARDTGRVDIMPTGDLDF	2-8*01	λ
20-7	1	3-9*01	3-22*01	4*02	AKDYTFEYDSSGYLDY	1-39*01	κ
20-16	8	4-39*01	3-16*0	3*02	ARHVVPLDQDYPTQTGAFDI	1-5*03	κ
20-8	66	3-9*01	3-22*01	5*02	AKGDSYDSSGYNPNWFDP	1-40*01	λ
20-20	49	3-9*01	3-22*01	5*02	ARGDSYDSSGYFPNWFDP		
20-2	1	1-18*01	3-22*01	4*02	ARDYYDSSPMGY	1-40*01	λ
20-1	27	1-2*02	6-19*01	4*02	ARDKGRVGESGGWYSFDY	3-21*01	λ
20-3	1	1-69*01	3-22*01	4*02	AGHLYYYDTSPLSI	2-14*01	λ
Total	1962						

Supplementary Figure 1. Summary of clones isolated from eighteen Ab-encoding libraries. The number of the clone, the germline V-, D-, and J-encoding region of the V_H-encoding gene, and the amino acid sequences of the respective CDR3 regions are shown. Representative clones analyzed in this study were highlighted in blue. An equivalent analysis of the germline genes in the V_L-encoding region of representative clones also is shown.
